# Small Molecule R1498 as a Well-Tolerated and Orally Active Kinase Inhibitor for Hepatocellular Carcinoma and Gastric Cancer Treatment via Targeting Angiogenesis and Mitosis Pathways

**DOI:** 10.1371/journal.pone.0065264

**Published:** 2013-06-05

**Authors:** Chao Zhang, Xihan Wu, Meifang Zhang, Liangcheng Zhu, Rong Zhao, Danqing Xu, Zhaohu Lin, Chungen Liang, Taiping Chen, Li Chen, Yi Ren, Joe Zhang, Ning Qin, Xiongwen Zhang

**Affiliations:** 1 Roche Research and Early Development China, Shanghai, China; 2 Crown Bioscience Inc. Beijing, China; Institut de Génétique et Développement de Rennes, France

## Abstract

Protein kinases play important roles in tumor development and progression. Lots of kinase inhibitors have entered into market and show promising clinical benefits. Here we report the discovery of a novel small molecule, well-tolerated, orally active kinase inhibitor, R1498, majorly targeting both angiogenic and mitotic pathways for the treatment of hepatocellular carcinoma (HCC) and gastric cancer (GC). A series of biochemical and cell-based assays indicated that the target kinase cluster of R1498 included Aurora kinases and VEGFR2 et al. R1498 showed moderate *in vitro* growth inhibition on a panel of tumor cells with IC50 of micromole range. The *in vivo* anti-tumor efficacy of R1498 was evaluated on a panel of GC and HCC xenografts in a parallel comparison with another multikinase inhibitor sorafenib. R1498 demonstrated superior efficacy and toxicity profile over sorafenib in all test models with >80% tumor growth inhibition and tumor regression in some xenogratfts. The therapeutic potential of R1498 was also highlighted by its efficacy on three human GC primary tumor derived xenograft models with 10–30% tumor regression rate. R1498 was shown to actively inhibit the Aurora A activity *in vivo*, and decrease the vascularization in tumors. Furthermore, R1498 presented good *in vivo* exposure and therapeutic window in the pharmacokinetic and dose range finding studies. Theses evidences indicate that R1498 is a potent, well-tolerated, orally active multitarget kinase inhibitor with a unique antiangiogenic and antiproliferative profile, and provide strong confidence for further development for HCC and GC therapy.

## Introduction

Protein kinases serve as targets for therapeutic intervention in cancers, which is validated and proved by the successful and broad application of protein kinase inhibitors in multiple cancers, either as single agent or in combination regimens. However, as a heterogeneous disease caused by accumulative multi-gene mutations rather than driven by single kinase mutant, cancers that hold good response to single agent therapy are very limited. In addition, the acquired resistance of tumors help themselves quickly evade from chemotherapy, then relapse. The complex aberrant signaling in cancers attracts the development of strategies that target multiple biological pathways relevant to tumor biology such as proliferation, metastasis and anti-apoptosis. One strategy involves rational drug combinations. For example, the combination of the VEGF targeted monoclonal antibody with conventional chemotherapy has demonstrated significant survival advantage in breast, colon, and lung cancers [1]. Another strategy is to develop the compounds that cover multiple mechanisms within a single agent. This approach has several potential advantages over combination strategies, including simplicity of the development path, speed to market, and less overlap of side effects. Currently, multikinase inhibitor sorafenib is used as first line therapy for advanced and metastatic HCC with improvement of the median survival time from 7.9 months (placebo group) to 10.7 months [2]. However, treatment with sorafenib results in statistically significant, but clinically mild, improvements in overall survival, time to progression and disease control rate [3]. Meanwhile, traditional cisplatin-based therapy is still widely used in clinical settings for advanced and metastatic GC. For HER2/neu overexpressing gastric adenocarcinomas, trastuzumab in combination with chemotherapy prolongs the median overall survival from 11.1 months (chemotherapy alone) to 13.8 months [4]. Although companioned diagnostic methodology has been established to screen target patients, trastuzumab has no activity in a large subset of patients harboring high level of HER2/neu with the reason to be identified [5]. Considering the high mortality of HCC and GC and current therapeutic regimens with limited outcome, there is still huge unmet medical need for both cancer types.

Angiogenesis based cancer therapy including anti-VEGFR-2 antibody, small molecules against VEGFR-2 signaling [6], [7], and VEGFR chimeric protein [8], has been proven to be an efficient strategy for treating of multiple cancer types. In addition, the efficacy of multikinase inhibitors sunitinib and sorafenib would partially be attributed to VEGF signaling blocking [9]. However, a number of patients are intrinsically resistant or develop resistance to anti-antiangiogenic therapy after several treatment cycles [10], [11]. Thus, clinical trials combining angiogenic inhibitors and drugs with alternative mechanism of action are expected to improve efficacy or overcome the resistance to antiangiogenic treatment [12]. It has been widely acknowledged that overexpression of aurora kinases in various cancers is involved in the process of tumorigenesis [13], [14]. Aurora kinase inhibitor VX-680 was able to effectively inhibit cancer cells growth *in vitro* and *in vivo*, and subsequently entered into clinical trials, suggesting that the Aurora kinases are druggable targets [15].

In light of the accumulative evidences of angiogenesis and Aurora kinases in cancer and their therapeutic values proven by the biological and small molecule inhibitors, we discovered a kinase inhibitor, R1498, which substantially affected the key pathways of angiogenesis and mitosis. R1498 has unique kinase inhibition profile, high potency, and good pharmacokinetic and toxicokinetic profiles, all these support further clinical development.

## Materials and Methods

### Ethics Statement

The use and care of experimental animals was approved by the Institutional Animal Care and Use Committee (IACUC), Roche R&D Center (China). Crown Bioscience, a CRO service company committed to protect patients’ privacy and to follow all the ethical principles for patient derived materials, collected freshly discarded human tumor samples, with IRB (local hospital equivalent committee) approval and patient consent requirements. All cell lines were purchased from ATCC, USA, or Shanghai Institutes of Biochemistry and Cell Biology, Chinese Academy of Science.

### Compounds

R1498 (purity >98%) was synthesized by Roche R&D Center (China). For enzymatic assays and *in vitro* cell based assays, R1498 was dissolved in DMSO as 0.01 mol/L stock solution. For animal studies, R1498 was dissolved in 1% Klucel EF/0.1% polysorbate 80/0.09% methylparaben/0.01% propylparaben water, the solution was prepared on a weekly basis. Sorafenib was synthesized by Roche R&D Center (China) and dissolved in cremophor EL/ethanol (50∶50, Sigma) to prepare a 5 mg/ml stock solution, foil wrapped, and store at room temperature. This stock solution was freshly prepared every 3 days. Final dosing solutions were prepared on the day of use by diluting the stock solution with sterilized water.

### Cell Lines

All cell lines from American Typical Collection Center (ATCC) and Cell bank, Shanghai Institutes of Biochemistry and Cell biology, Chinese Academy of Sciences were maintained at 37°C with 5 % CO_2_ humidified atmosphere in growth medium recommend by the vendors and subjected to *in vitro* assays between passages 8∼15, the cell lines for animal studies were between passages 5∼10. Human umbilical vein endothelial cell (HUVEC) obtained from Allcells (Emeryville, CA) was kept in EGM-2 (LONZA, Allendale, NJ) with endothelial cell growth supplements and 10% fetal bovine serum (Invitrogen, Carlsbad, CA).

### Cell Proliferation Assay

Each cell line was seeded in a 96-well tissue culture plate (Corning, NY) at a predetermined density in 180 µL complete medium, attached overnight and treated by compound for another 72 h. Then the medium was discarded and replaced with 10% CCK-8 (Dojindo, Kumamoto, Japan) in complete medium, then incubated the plates for another 2 h. The OD_450_ was measured with SpectraMAX 190 spectrophotometer (MDS, Sunnyvale, CA). A background absorbance of OD_blank_ was subtracted from all wells. Then inhibition rate (IR) was determined with following formula: IR (%) = (OD_DMSO_−OD_compound_)/OD_DMSO_×100%.

In VEGF-induced HUVEC proliferation assay, the HUVECs suspended in 170 µL EGM-2 with 0.5% FBS were seeded and incubated overnight. After the cells attached, 10 µL 1 µg/ml recombinant human VEGF 165 (R&D systems, Minneapolis, MN) was added to each well together with 20 µl compound solutions prepared in EGM-2 with 0.5% FBS. The CCK-8 assay as above was used for determination of cell viability.

### Kinase Assays

The affinity of R1498 against 402 kinases was determined by KINOMEScan® (Ambit Biosciences, San Diego, CA) and data were presented as dissociation constant (Kd) values[16]. The inhibition on kinase activity of Aurora kinases and VEGFR2 was further characterized by Z’-Lyte kinase activity assays under corresponding ATP concentrations.

### HUVEC Tube Formation Assay

HUVEC tube formation assay was carried out as previously reported [17]. Briefly, Matrigel™ (BD Biosciences, Franklin Lakes, NJ) was thawed at 4°C for 3∼4 h. 100 µl liquid Matrigel™ was added to pre-cooled 96-well tissue culture plate, and the plate was incubated at 37°C, 5 % CO_2_ humidified atmosphere for 1 h to solidify Matrigel™. The HUVECs were cultured in EGM-2 with 0.5% FBS overnight, and seeded in 90 µL EGM-2 with 0.5% FBS at a density of 2.2×10^5^ cells/ml on the Matrigel™ coated plate. The cells were treated by compound or equivalent amount of DMSO. The plate was incubated at 37°C, 5 % CO_2_ humidified atmosphere for 7 h, then cell morphology was captured with phase-contrast microscope (IX71, Olympus, Hamburg, Germany).

### Western Blot

Cell lysate was prepared in RIPA buffer and quantified by the bicinchoninic acid (BCA) method (Pierce, Rockford, IL). Thirty micrograms of protein per sample was loaded onto a 4∼12% NuPAGE® Novex SDS gel (Invitrogen). The protein was transferred by an iBlot® dry blotting device (Invitrogen) onto nitrocellulose membranes. After blocking nonspecific binding with TBS/Tween20 (0.1%) (TBS/T) containing 5% non-fat milk for 1 h at room temperature, the membrane was incubated in phospho-Aurora A (Thr288) (C39D8) rabbit monoclonal antibody (Cell Signaling Technology, CST #3079, Beverly, MA) or phospho-Histone H3 (Ser10) rabbit polyclonal antibody (CST#9701) (1∶1000 in TBS/T containing 3% bovine serum albumin (BSA), and gently shaken at 4°C overnight. The membrane was washed with TBS/T three times to remove the unbound antibody, then incubated with the secondary antibody (HRP-conjugated goat anti-mouse IgG or goat anti-rabbit IgG, 1∶10000, KangChen Biotech, Shanghai, China) for 1 h at room temperature, respectively. Protein bands were visualized with an enhanced chemiluminescence (ECL) kit (Pierce, Rockford, IL).

### Immunofluorescence

Cells seeded on chamber slides were fixed in 4% paraformaldehyde at room temperature, then permeabilized with 0.15% Triton-X100 in TBS and blocked in 3% BSA in TBS/T. The primary antibodies (phospho-Aurora A (Thr288) (CST#3097), phospho-Histone H3 (Ser10) (CST#9701) and MPM2 (anti-phospho-Ser/Thr-Pro) Millipore #05-368) were incubated with slides in a wet chamber at 4°C overnight. The secondary antibodies Alexa Fluor® 488 goat anti-rabbit IgG (H+L) or Alexa Fluor® 594 goat anti-mouse IgG (H+L) (Invitrogen) were applied respectively for one hour after the slides were washed with TBS thoroughly. After the unbound antibodies were removed, the slides were dried and mounted with DAKO antifide mounting medium. The images were captured with IX71 under appropriate excitation and emission filters.

### Flow Cytometry

DNA content was measured with a FACS-Calibur cytometer (Becton Dickinson, San Jose, CA) and cell cycle distribution was calculated as described previously [18].

### Tubulin *in vitro* Polymerization Assay

*In vitro* tubulin polymerization assay was performed as described previously [18].

### Single Dose Pharmacokinetic (SDPK) Study

The use and care of experimental animal was approved by Institutional Animal Care and Use Committee, Roche R&D Center (China). Male nude mice (body weight: 19 to 24 g) were used in SDPK study. Before the pharmacokinetic study, animals were randomly assigned to the sampling groups (3 animals per time point). Ten additional mice were used to collected plasma blank for calibration curves. The mice were fasted overnight before oral administration.

Blood samples were collected by retro-orbital puncture at pre-dose and 0.033, 0.083, 0.25,0.5, 1, 2, 4, 6, 8, and 24 hours post-dose. The plasma samples and the dose formulation were stored at −20°C until bioanalysis. The data acquisition and control system were created using Analyst 1.4 software from ABI Inc. The concentrations in plasma below the limit of quantitation (LOQ = 1 ng/mL) were designated as zero. The pharmacokinetic data analysis was performed using noncompartmental analysis modules in WinNonlin Professional v5.2 (Pharsight, USA).The bioavailability was calculated as F (%) = (Dose_iv_×AUC_po(0-∞)_)/(Dose_po_×AUC_iv(0-∞)_)*100%. For rat, dog and monkey SDPK, the blood samples were collected from jugular vein puncture.

### Xenograft Efficacy Study with Cell Lines and Patient Derived Tumors

The use and care of experimental animal was approved by Institutional Animal Care and Use Committee, Roche R&D Center (China). Tumor cells were inoculated into the right flank of BALB/C *nu/nu* female nude mice (5∼6 weeks, 16∼18 g, obtained from Sino-British SIPPR/BK Lab. Animal Ltd, Shanghai, China) at optimal density based on our in-house validation study. After the tumor was established and reached 100∼150 mm^3^, the mice were randomized into ten mice per group and received treatment according to the schedule. The tumor growth was recorded three times a week with the measurement of length (L) and width (W) by caliper, and calculated as tumor volume (TV, mm^3^) = 0.5*L*W*W (mm). The tumor growth inhibition was calculated as TGI%  = (1−(mean tumor volume of the treatment group on the first day-mean tumor volume of the treatment group on the end day)/ (mean tumor volume of the control group on the first day-mean tumor volume of the control group on the end day))×100%. The tumor regression of individual mouse was defined - the tumor volume at the end was less than the tumor volume when treatment was initiated.

In human primary tumor models, fresh human gastric tumor tissues were directly implanted into nonobese diabetic and severe combined immune-deficient (NOD-SCID) mice within 5 hours post-surgery with 5 mm^3^ fragments to establish primary tumor xenograft models. Each tumor model was verified by at least three serial *in vivo* passages to demonstrate engraftment stability. Tumors from passages 4–7 were subcutaneously inoculated with trocars into right flanks of BALB/C nu/nu female nude mice (5∼6 weeks, 16∼18 g). Treatment began when tumor volume reached 100∼150 mm^3^. The tumor measurement and operation protocol was the same as cell line derived tumor model.

### Immunohistochemistry

The 8 µm paraffin embedded tumor tissue slides prepared from tumor of *in vivo* efficacy studies were heated in a dry oven at 60°C for 1 hour, then deparaffinized and hydrolyzed by Leica autostainer XL ST5010 at room temperature. Antigen retrieval was carried out at 95∼99°C in citrate buffer (0.01 M, pH 6.0) heated by a medical microwave at 92–98°C for 5 min, then cooled down to room temperature. Then peroxidase-blocking reagent (DAKO, DK-2600, Glostrup Denmark) was applied to quench the endogenous peroxidase. The slides were incubated in blocking serum for 20 min, then covered by 100 µL phospho-Aurora A (Thr288) (C39D8) rabbit mAb (CST #3079, 1∶200 in TBS/T) or rabbit anti-human CD31 polyclonal antibody (Santa Cruz Biotech, sc-8306, Santa Cruz, CA, USA; 1∶200 in TBS/T) at 4°C overnight, then incubated in 100 µL DAKO Envision+/HRP, rabbit/mouse after being washed thoroughly with TBS. One hundred micro liters of substrate-chromogenic solution (DAB) were applied to the slides and incubated at room temperature for 10 min. The slides were rinsed gently with tap water for 15 min. Counterstain and dehydration was performed by XL ST5010 at room temperature. Finally, the slides were mounted with Permount mounting medium (Fisher Scientific, Miami, FL).

### Dose Range Finding and Toxicokinetic Study

Dose range finding and toxicokinetic study was carried out in rats and Beagle dogs based on *in vitro* metabolites identification. Wistar rats (male: 3, female 3 in each group, from Vital River, Beijing, China) were fasted overnight, and then received test articles as 0, 6.25, 50 and 100 mg/kg twice a day per oral gavage for a 14-day continuous regimen. Motility observation was applied on a daily bases to find the maximum tolerant dose for the animals. The animals were sacrificed on d14, and the following toxicology observation were carried out including body weight changes, clinical observation, gross necropsy, hematology chemistry and histopathology. On day 1 and day 14, blood samples were obtained via jugular vein puncture from all animals (if alive, using EDTA-K2 tubes). Samples were mixed gently and placed on crushed wet-ice until centrifugation, which was carried out immediately. The samples were centrifuged for approximately 15 minutes at 4°C 2,700 rpm. The resultant plasma was separated, transferred to uniquely labeled clear polypropylene tubes and frozen immediately over solid carbon dioxide (dry ice) until transferred to approximately −80°C. Each animal was bled at the following time points: approximately 5 min, 15 min, 30 min, 1, 2, 4, 8 and 24 hours post first dose on the specific day. The drug concentrations in the plasma were determined by LC-MS/MS. The pharmacokinetic analysis was performed using WinNonlin software (Version 5.2, Pharsight Corporation, CA, USA), and the pharmacokinetic parameters were calculated using a Non-compartmental model method.

Beagle dogs (approximately 8–10 months, from Beijing Marshall Biotechnology Co. Ltd., Beijing, China) were subjected to the study protocol as described above. Each group has one male and one female dog, the dosages of test article were: 0, 1, 7.5 and 15 mg/kg, respectively. The use and care of experimental animal was approved by Institutional Animal Care and Use Committee, Roche R&D Center (China).

### Data Analysis and Statistics

Independent t test was used for continuous data analysis, and χ2 test was applied to categorical data. Data were considered significant when p values were <0.05.

## Results

### 1. Characterization of R1498 as a Multikinase Inhibitor

**Figure 1:**
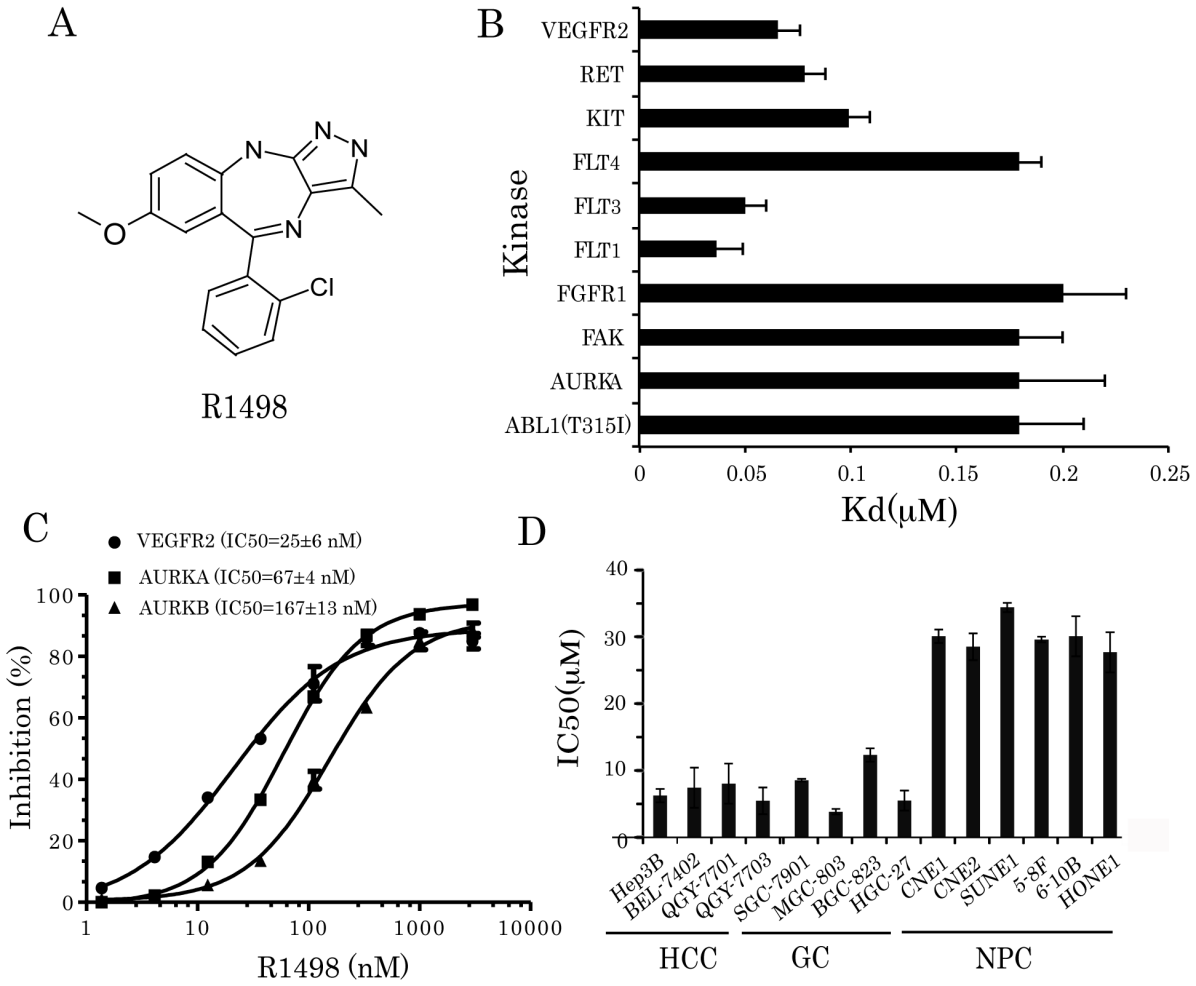
*In vitro* activity of R1498. (**A**) Chemical structure of R1498; (**B**) R1498 was subjected KINOMEScan® and the most potent hits with Kd< 0.2 µM are shown; the assay was carried out in triplicate, and data were presented as mean±standard deviation; (C) R1498 was tested in Z-lyte kinase assays including VEGFR2, Aurora A and B, the dose response curve was shown and mean IC50 values from three independent experiments were indicated. (D) The *in vitro* anti-tumor growth activity of R498 was assayed on 16 cell lines including HCC, GC and NPC. Data from triplicated independent assays were presented as mean±standard deviation.

Pyrazolobenzodiazepine analog R1498, with a scaffold based on the fusion of a benzodiazepine and pyrazole ring (Figure 1A), was previously identified to be a weak cyclin-dependent kinase 2 (CDK2) inhibitor [19]. This molecule showed the nature of a multikinase inhibitor when we analyzed the kinase inhibitor library. The kinase inhibition profile of R1498 (1 µM) was characterized via KINOMEScan® at ATP concentration around the Km of each kinase. The inhibition rate of R1498 is over 80% against 39 kinases out of 402 kinases, including 359 wild-type kinases and 43 kinase mutants (Figure S1). The dissociation constants (Kd) of 39 hit kinases were then determined, and the kinases with Kd <0.2 µM were shown in Figure 1B. Most of the potential hits belong to the tyrosine kinase (TK) family and AGC family. In addition, Z-lyte assay was carried out for further determination of the kinase inhibition activity of R1498. The results showed that IC50s of R1498 were 25±6, 67±4, 167±13 nM against VEGFR2, Aurora kinase A and B, respectively (Figure 1C). Then our study was focused on the anti-angiogenic and anti-mitotic pathways majorly affected by R1498.

A panel of 16 cancer cell lines, including hepatocarcinoma, gastric cancer and nasopharyngeal carcinoma cell lines, was used to determine the *in vitro* anti-proliferation ability of R1498. Most of the cell lines are established from Asian cancers, as the Asian prevalent cancers are our focus. The hepatocarcinoma and gastric cancer cell line panel were more sensitive to R1498 treatment than nasopharyngeal cancer cell lines. The overall mean IC50s were 7.81, 7.55 and 30.07 µM, corresponding to epatocellular carcinoma, gastric cancer, and nasopharyngeal carcinoma cell lines, respectively. The sensitivity of cell lines derived from Asian population (BEL-7402, QGY-7701 and QGY-7703) was comparable to the sensitivity of Hep3B that was from Caucasian (Figure 1D).

### 2. R1498 Inhibits Aurora Kinases and Induces Cell Cycle Arrest

**Figure 2:**
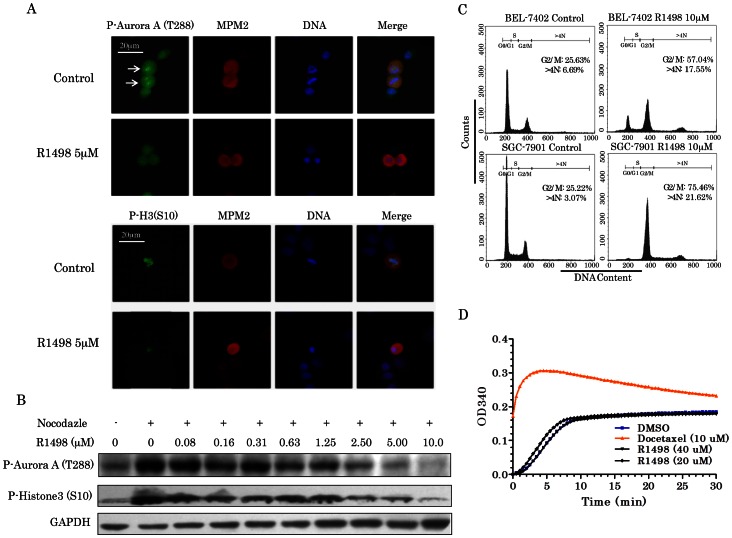
R1498 targets Aurora kinases in cells. (**A**) SGC-7901 gastric cancer cells were treated with 5 µM R1498 for 24 h, then fixed and stained for phospho-Aurora A(T288) (green), MPM2 (red) and DNA (blue). MPM2 was used to locate the mitotic cells. Bright green foci pointed by white arrow heads indicated the high level of phospho-Aurora A(T288) in centromere (upper panel). R1498 treated SGC-7901 gastric cancer cells as above were stained for phospho-Histone H3(H10) (green), MPM2 (red) and DNA (blue) (lower panel). Representative images from 2 independent assays are shown. (**B**) R1498 treated SGC-7901 cells as above was subjected DNA analysis by flow cytometry via propidium iodide staining. The histograms were further analyzed by Modfit 3.0 for cell cycle distribution. The cells with DNA content more than 4N (>4N) mean aneuploidy caused by endoduplication. Representative histograms from 3 independent assays are shown. (**C**) SGC-7901 synchronized with nocodazole (300 nM) for 12 h were treated with various concentration of R1498, then lysed and subjected for western blot with corresponding antibodies. (**D**) *In vitro* tubulin polymerization assay was performed with R1498 and docetaxel, and the OD340 was measured and recorded in a time lapsed manner. Representative histograms from 2 independent assays are shown.

Aurora kinase inhibition by R1498 was visualized by immunofluorescence in gastric cancer cell line SGC-7901. Direct phosphorylation sites phospho-Aurora kinase A (T288) and phospho-histone H3 (S10) were used to indicate the kinase activity of Aurora A and B, respectively [20], [21]. Phospho-Ser/Thr-Pro Mitotic protein monoclonal #2 (MPM2) was used as a mitotic marker (red) for labeling mitotic cells. Aurora kinase A (T288) phosphorylation occurs in the centrosome of mitotic cells (green). After the cells were treated with 5 µM MR1498 for 24 h, the Aurora A T288 green foci in mitotic cells became weaker or disappeared (Figure 2A, upper panel),showing that the activity of Aurora kinase A decreased upon R1498 treatment. The phosphorylation of histone H3 (S10) is mainly localized in the centromeres of mitotic cells. After the cells were treated with R1498, the phospho-histone H3 (S10) decreased sharply (green). This observation suggested that the activity of Aurora kinase B also dropped down upon R1498 treatment. These results from immunofluorescence were confirmed by western blot. As shown in Figure 2B, the phosphorylation of phospho-Aurora kinase A (T288) and phospho-histone H3 (S10) decreased in a concentration-dependent manner in acute treatment by R1498.

Inhibition of Aurora kinases A and B produces different phenotypic changes in cell cycle. Suppression of Aurora A induced cell cycle G2 phase arrest [22], while inhibition on Aurora kinase B is reported to cause polyploidy in cells due to endoduplication without cytokinesis[15]. After the cells were treated with 10 µM R1498, both SGC-7901 (gastric cancer cell) and BEL-7402 (hepatocellular carcinoma cell) showed G2/M phase arrest and accumulation of polyploid cells. For BEL-7402, the cells distributing in G2/M phases increased from 25.6% to 57.0%, meanwhile, the cells with >4 N DNA content increased from 6.7% to 17.6%. SGC-7901 was more sensitive, with the G2/M population increased from 25.2% to 75.5%, and polyploid cells increased from 3.1% to 21.6% (Figure 2C). Microtubule stabilizer (for example taxol) and destabilizer (for example colchicine) will cause G2/M phase arrest as well. To understand whether R1498 is a microtubule targeted reagent, *in vitro* tubulin polymerization assay was carried out. The data showed that R1498 neither stabilized nor destabilized microtubule polymerization even under high R1498 concentration (40 µM) *in vitro* (Figure 2D), suggesting that the cell cycle G2/M arrest was not caused by microtubule cytoskeleton disturbance. Taken together, these data suggested that R1498 inhibited the Aurora kinases A and B in cells and produced the phenotypic changes in cells.

### 3. R1498 Antagonizes the Angiogenesis Progress of Human Endothelial Cells

**Figure 3:**
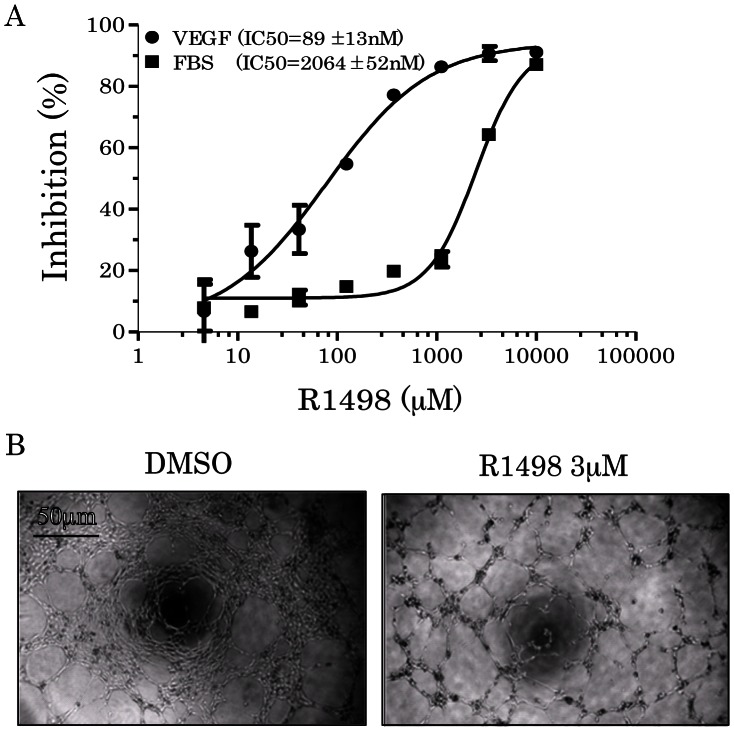
R1498 targets VEGF signaling in cells. (**A**) HUVECs cultured in 10% FBS, or 0.5% FBS with VEGF 165 were treated with various R1498 for 72 h, and then analyzed for proliferation endpoint. The curves were fitted with Graphpad Prism5.0. The three independent assays were carried out in triplicated and IC50s were indicated as mean±standard deviation; (**B**) HUVECs grown on Matrigel™ were treated with 3 µM R1498 or equivalent DMSO for 8 h. Representative images from 2 independent assays are shown.

Angiogenesis was initialized by secretion of angiogenic growth factor from tumor cells, and then the endothelial cells proliferate, migrate and form vessel tubes in responses to the VEGF stimulation. To address the specificity of R1498 antagonizing the endothelial cell growth stimulated by VEGF, human umbilical vein endothelial cells (HUVECs) were exposed to two different stimuli, VEGF and FBS, under the treatment of R1498 for 72 h. Under the culture conditions containing VEGF or FBS, HUVECs showed different response to R1498 . The IC50s were 89 and 2064 nM for VEGF and FBS, respectively; these suggested that R1498 antagonized the HUVEC growth driven by VEGF more potent than by FBS (Figure 3A). In HUVEC tube formation assay, after 8 hr exposure to R1498, HUVECs formed fewer intact tube meshes than DMSO treated groups (Figure 3B). R1498 didn’t affect HUVEC proliferation under this treatment condition (data not shown).

### 4. R1498 Suppresses the Growth of Human Cancer Xenografts

**Figure 4:**
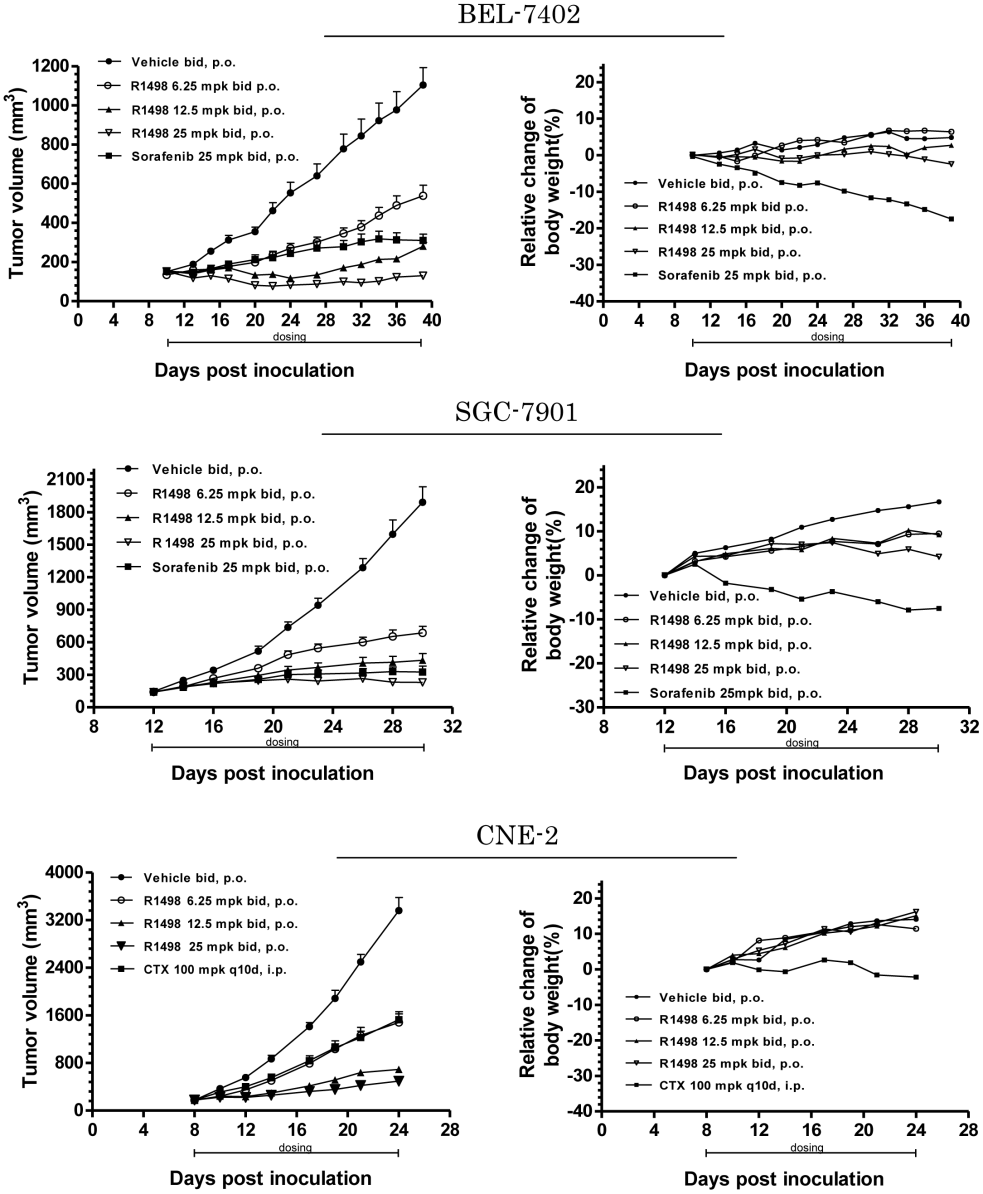
*In vivo* efficacy of R1498 on cell line derived xenografts. BEL-7402, SGC-7901 and CNE-2 xenograft bearing nude mice were treated with 25 mg/kg R1498 or sorafenib (or cyclophosphamide (CTX)) as indicated schedule; the tumor volume was recorded and plotted against days post inoculation (upper panel), the body weight changes compared with the day of starting treatment was plotted (lower panel). The volume and relative body weight changes were indicated as mean±standard error (n = 12 in vehicle groups, n = 10 in R1498/sorafenib/ cyclophosphamide groups). The studies were repeated for confirmation twice. The representative curves are shown here.

The pharmacokinetic properties with single dose of R1498 in multiple species were determined. The half-life time (T1/2) and oral bioavailability (Oral BA%) favors further efficacy study in nude mouse (Table S1) with a twice-daily oral gavage schedule. A panel of xenografts including Asian gastric cancer, hepatocarcinoma and nasopharyngeal cancer was employed for comparison between R1498 and another multikinase inhibitor sorafenib (Figure 4, Table S2). Sorafenib is approved as first line therapy for hepatocarcinoma [2], and is in clinical trial for combinatorial therapy for advanced gastric cancer [23]. The parallel comparison consisted of tumor growth inhibition, regression and body weight changes of animal with dosing of 25 mg/kg, twice daily per oral. In all tested xenografts, R1498 showed better tumor growth inhibition rate (TGI%) over sorafenib. For hepatocarcinoma, the TGI% of R1498 is 10–19 % more than TGI% of sorafenib, for gastric cancer, 2–12% over TGI% of sorafenib . The regression rate of tumor was also incorporated as another therapeutic endpoint. In hepatocarcinoma panel, tumor regression was observed in BEL-7402 model, 8/10 (80%) animals had regression in R1498 group, while 1/10 (10%) animal had regression in sorafenib group (Figure 4, BEL-7402). For gastric cancer panel, MGC-803 and SGC-7901 xenografts had the same regression rate in response to the test articles: 5/10 (50%) for R1498, and 2/10 (20%) for sorafenib (Figure 4, SGC-7901; and Table S2). Based on the efficacy endpoints from all 7 tested models, R1498 has better efficacy than sorafenib under the same treatment schedule. Moreover, R1498 showed good efficacy on one nasopharyngeal carcinoma xenograft, the TGI% of R1498 (90%, 25 mg/kg, twice-daily, per oral gavage) was superior over standard of care- cyclophosphamide (60%, every 10 days, intraperitoneal injection) (Figure 4, CNE-2). The body weight changes which were recorded in all models for toxicity comparison showed that the nude mouse showed more tolerable to R1498 during the entire treatment in BEL-7402, SGC-7901 and CNE-2 models, although body weight gain decreased slightly 10 days after treatment (Figure 4, Table S2). However, sorafenib caused continuous drop of the animal body weight. For sorafenib treated mouse in BEL-7402 and SGC-7901 models, the percentage of body weight loss exceeded 15% on the termination day. As shown in Table S2, R1498 showed less impact on mouse body weight than sorafenib did, which suggested that R1498 held better safety profile.

### 5. R1498 Down-regulates Phosphorylation of Aurora A and Vascular Density in Tumor

**Figure 5:**
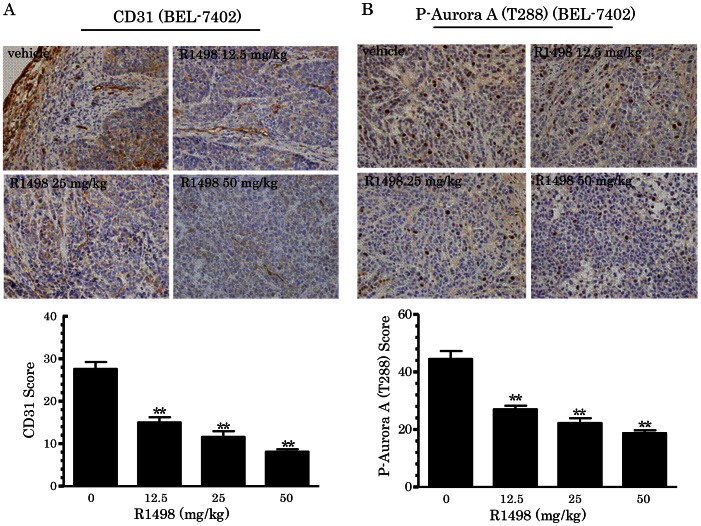
R1498 antagonized angiogenesis and Aurora A activity *in vivo*. The tumors harvested from said BEL-7402 study (n = 12 in vehicle groups, n = 10 in R1498/sorafenib/CTX groups) were sectioned and subjected for immunohistochemistry of CD31 (angiogenesis marker, A) and phosphor-Aurora A(T288) (Aurora kinase A activity marker, B) staining. The slides were reviewed and scored by two independent pathologists. The scores were presented as mean±standard error. (** p< 0.01; Log-rank test).

Phosphorylated Aurora A (T288) and CD31 in tumors from efficacy study were used for determination of the on-target effect of R1498. The tumor mass harvested from the BEL-7402 xenograft study was prepared into paraffin embedded slides and subjected to immunohistochemisty for visualizing angiogenesis maker human CD31 and Aurora kinase A activity marker phospho-Aurora A (T288). In BEL-7402 tumors, the CD31 staining of R1498 treatment groups decreased in a dose-dependent manner, which suggested the vascularization in tumors was inhibited (Figure 5A). The activity of Aurora kinase A was also reduced upon R1498 treatment, which was reflected by the weaker staining of phospho-Aurora A (T288) in R1498 treated tumors (Figure 5B).

**Figure 6:**
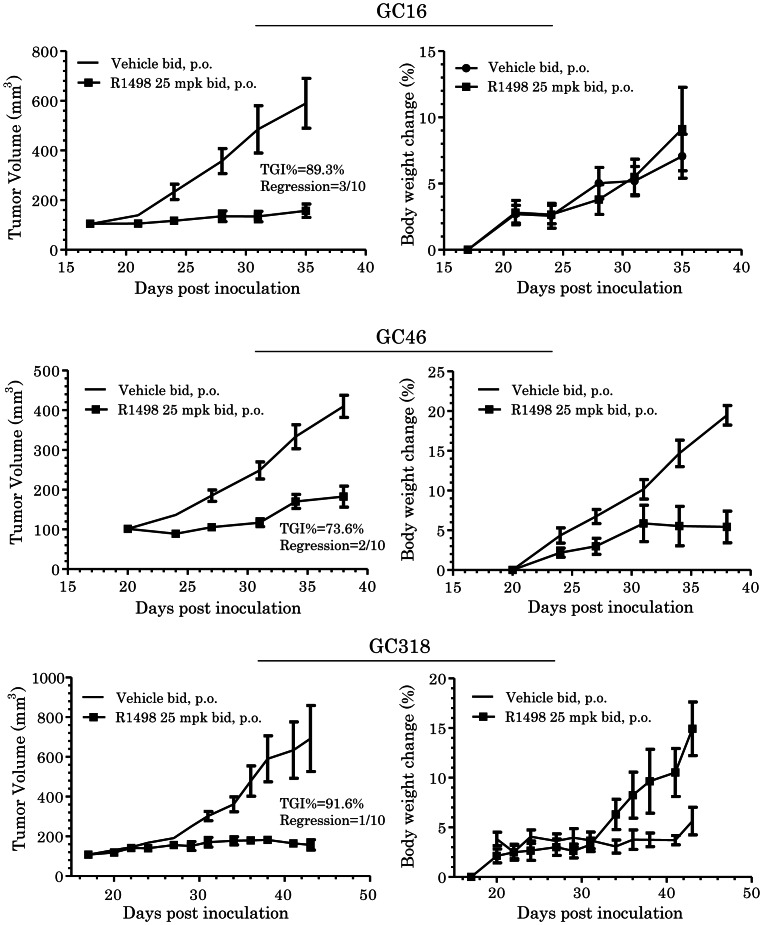
*In vivo* efficacy of R1498 on patient primary tumor derived xenografts. R1498 was administrated to nude mice bearing xenografts derived from three different gastric cancer patients as indicated schedule, the tumor volumes were recorded and plotted against days post inoculation (left panel), together with the body weight changes (right panel). The volume and relative body weight changes were indicated as mean±standard error (n = 10).

Human primary gastric tumor derived xenograft model was used to predict the efficacy for clinical translation. The cancerous tissues of three individual gastric cancer patients were engrafted in mouse, and xenografts delineated different growth curves *in vivo*. The xenografts showed different impact on the body weight changes of the hosts. The mice were treated with R1498 (25 mg/kg) for the indicated period, the final TGI% reached 73.6 ∼ 91.6%,with regression rates 10∼30%. The limited body weight changes suggested that the tumor bearing mice were well tolerant to R1498 (Figure 6).

### 6. R1498 has Good Pharmacokinetic Profile (PK) and Therapeutic Window

SDPK indicated that the acceptable bioavailability and half-life properties of R1498 among nude mouse, rat and dog (Table S1). Pooled liver microsomes were used to predict the major metabolites in different species. Rat and beagle dog with similar microsome metabolites of human were used in maximum tolerant dose (MTD) study to determine the therapeutic window between efficacious and toxic exposures (Table S4). Female and male rats showed different response to the escalated doses of R1498. Female rats began to lose body weight at 12.5 mg/kg, one female rat died at 50 mg/kg (MTD); all male rats behaved normally at 50 mg/kg, but began to lose weight at 100 mg/kg (MTD>100 mg/kg), the corresponding area under curve (0–24 h) (AUC_0–24_) was 211000 (female) and >155000 (male) ng·hr/ml. The ED60 AUC_0-24_ estimated from pharmacokinetic study of BEL-7402 model was 5000–10000 ng·hr/ml, the ED90 AUC_0–24_ was ∼26000 ng·hr/ml (Table S3). So the ratio between MTD AUC and ED60 AUC were 21–42 (female), or over 15–31 (male); the ratio between MTD AUC and ED90 AUC were 8.1 (female) or over 6.0 (male). Following the same procedures, the female dog began to lose weight at 7.5 mg/mg, and all the dogs kept alive at the highest dose (15 mg/kg), thus the MTD for beagle dogs was over 15 mg/kg, the MTD (0–24 h) (AUC_0–24_) are over 111000 ng·hr/ml, based on these parameters, the ratio between MTD AUC and ED60/ED90 AUC were over 11–22/>4.3, respectively (Table S5). The dose range finding study predicted the efficacious exposure far below toxic exposure, suggesting good therapeutic window for R1498.

## Discussion

R1498 was discovered as a kinase inhibitor for the treatment of hepatocellular carcinoma (HCC) and gastric cancer (GC). In this research, we have characterized the unique mechanism of action of this small molecule *in vitro* and *in vivo*, as well as determined its pharmacokinetic and toxicology profiles, to address the efficacy and safety profile of R1498 as an antitumor agent.

The major targets affected by R1498 are mitotic kinases and angiogenic receptor tyrosine kinases, especially Aurora kinases and VEGFR2, with corresponding phenotypic changes (Figure 1). The kinase inhibition activity of R1498 was moderate compared with other multikinase inhibitor. For example, sorafenib has IC50s of 7 nM for Raf-1, 22 nM for b-Raf; ENMD-2076 has IC50 s of 14 nM for Aurora A, 1.86 nM for Flt3, 58.2 nM for VEGFR2; regorafenib has IC50s of 4.2 nM for VEGFR2, 7 nM for KIT, 1.5 nM for Ret; R1498 has no single digit nanomole IC50 on any tested kinase. This moderate kinase inhibition was translated into micromole level IC50 in cell proliferation inhibition, which is weaker than sorafenib, ENMD-2076, and regorafenib [24], [25], [26]. Among the mentioned multikinase inhibitors above, ENMD-2076 shares similar mechanism of action with R1498. ENMD-2076 is a novel vinyl pyrimidine chemical entity actively against Aurora A and angiogenic kinases including VEGFR2, FLT3 et al. ENMD-2076 inhibited the *in vitro* growth of colorectal cancer, breast cancer, lung cancer, prostate cancer and leukemia cell lines with better IC50s of about hundreds nanomole [25]. The IC50 of ENMD-2076 on Aurora A was 14 nM, which is more potent than R1498 (IC50 = 67 nM on Aurora A), which is possibly the reason why in vitro potency on cell proliferation of ENMD-2076 was better than R1498. In VEGF driven HUVEC proliferation assay, ENMD-2076 and R1498 have comparable efficacy. In the xenografts panel, ENMD-2076 inhibited the growth of colorectal cancer, breast cancer, melanoma, and leukemia xenografts with TGI% ∼90 at the dose of 150∼225 mg/kg once daily per oral; the regression was observed in HCT-116 and MDA-MB-231 models at 200 mg/kg twice daily/once daily schedule. R1498 achieved similar efficacy at 25 mg/kg twice daily schedule without observed toxicology signs. The data suggested R1498 achieved much better efficacy *in vivo* although it was a little weaker than ENMD-2076 *in vitro.* The superior *in vivo* efficacy of R1498 may come from better VEGFR2 inhibition over ENMD-2076 (25 nM vs 58 nM of IC50 from biochemical assay), and could also be the different PK properties of the two compounds, to be specific, the different bioavailability. We have compared the 50 mg/kg qd R1498 with 25 mg/kg bid schedules, the results showed 25 mg/kg bid achieved better efficacy. However, ENMD-2076 has similar efficacy with 100 mg/kg qd and 50 mg/kg bid schedules in MV4; 11 model, which suggested ENMD-2076 had reasonable half-life in nude mice [25], thus the bioavailability of R1498 could be on reason for better efficacy at lower dose. Taken together, the superior in vivo efficacy of R1498 over ENMD-2076 possibly owns to its ideal bioavailability and better VEGFR inhibition.

In this study, we carried out side-by-side comparison of the *in vivo* efficacy between R1498 and sorafenib. Both compounds held remarkable *in vivo* anti-tumor efficacy, even induced tumor regression in some xenografts. R1498 has better efficacy but less toxicity than sorafenib in most of the test models. The target clusters of sorafenib (receptor tyrosine kinases and c-raf) [24] are dissimilar with R1498, which possibly is one of the reasons for their diversified toxic profiles. A major expectation for targeted molecular cancer therapeutics is that they show good efficacy and low toxicity. However, it still remains to be addressed that how to balance the toxicity of targeting multiple kinases and efficacy in multikinase inhibitor discovery even though there are several successful role models already [27], [28], [29]. The effect of multikinase inhibitors is broad, and possibly less problematic than one might have envisioned regarding efficacy, but it’s hard to predict how useful such inhibitors will be in clinical settings. Whether broad spectrum, multikinase inhibitors or highly selective second and third generation kinase inhibitors will ultimately be more efficacious and safe remains to be established. Non-selective kinase inhibitor (Sunitinib) showed good efficacy but comparatively high toxicity profile [30]. The kinase selectivity profile of R1498 favors the balance between drug efficacy and toxicity. Compared with other multikinase inhibitors, although the inhibition rate of R1498 is over 80% against 39 kinases out of 402 kinases at 1 µM, and the IC50s of R1498 on the hit kinases are moderate, targeting mitosis and angiogenesis kinase panel induces significant tumor growth inhibition with well-tolerated property.

Two important components of the six hallmarks of cancer are “inducing angiogenesis” and “enabling replicative immortality” [31]. Considering mitosis of cancer cells and angiogenesis by endothelial cells are two key steps of tumorigenesis, we believe that the therapeutic strategy of targeting these two types of cells (both tumor cells and tumor vascular cells) may be of important value in cancer therapy. Simultaneous inhibition of VEGF and Aurora signaling while retaining overall kinases selectivity may provide a therapy with improved efficacy and less resistance. Dual-pathway (tumor proliferation and angiogenesis) kinases inhibitor may show improved antitumor activity over drugs that target either one of these targets/pathways alone.

We here are trying to identify novel therapeutic solutions for cancers prevalent in Asian population. Our research indicates that Aurora kinase and VEGFR2 inhibitor R1498 shows on-target phenotype in Asian HCC and GC cell and in animal models, and produces significant tumor growth inhibition *in vivo*, of special note, in human primary derived xenografts with ideal pharmacokinetic profile and therapeutic window. The concept of targeting mitosis and angiogenesis pathways in parallel may bring novel Asian HCC and GC therapeutic approaches for development of multitarget kinase inhibitor, as well as for combinatorial therapeutics.

## Supporting Information

Figure S1 **Treespot map of R1498 (1 µM) under ATP Km.** The affinity of R1498 against 402 kinases was determined by KINOMEScan® (Ambit Biosciences, San Diego, CA). The inhibition was indicated with red dots of different size in a kinome tree. Bigger dot means strong inhibition.
(DOC)

Table S1 Single dose PK profiles in multiple animal species. R1498 micronized active pharmaceutical ingredient (API) was formulated and dosed to multiple species per oral or intravenous injection. Plasma samples from various time points were collected and determined for R1498 concentration.
(DOC)

Table S2 Efficacy of R1498 and sorafenib or cyclophosphamide on xenograft models of Chinese cancers. R1498, sorafenib and cyclophosphamide were tested on multiple xenografts with indicated schedules.
(DOC)

Table S3 ED60/90 estimation from the R1498 exposure at steady state in tumors. The exposure in plasma and tumor at steady state were plot against tumor growth inhibiton rate to estimate the ED60/E90 doses.
(DOC)

Table S4 Metabolite profiles in pooled liver microsomes. Pooled liver microsomes were incubated with R1498 and the metabolites were identified to predict the most close species to human.
(DOC)

Table S5 Dose range finding (DRF)-tox and efficacious exposures of R1498.
(DOC)
