# Supplementary figures and images for: Small Molecule R1498 as a Well-Tolerated and Orally Active Kinase Inhibitor for Hepatocellular Carcinoma and Gastric Cancer Treatment via Targeting Angiogenesis and Mitosis Pathways
Source: PLoS One. 2013 Jun 5;8(6):e65264. doi: 10.1371/journal.pone.0065264 (PMC3673949; doi:10.1371/journal.pone.0065264)

**Figure S1.**


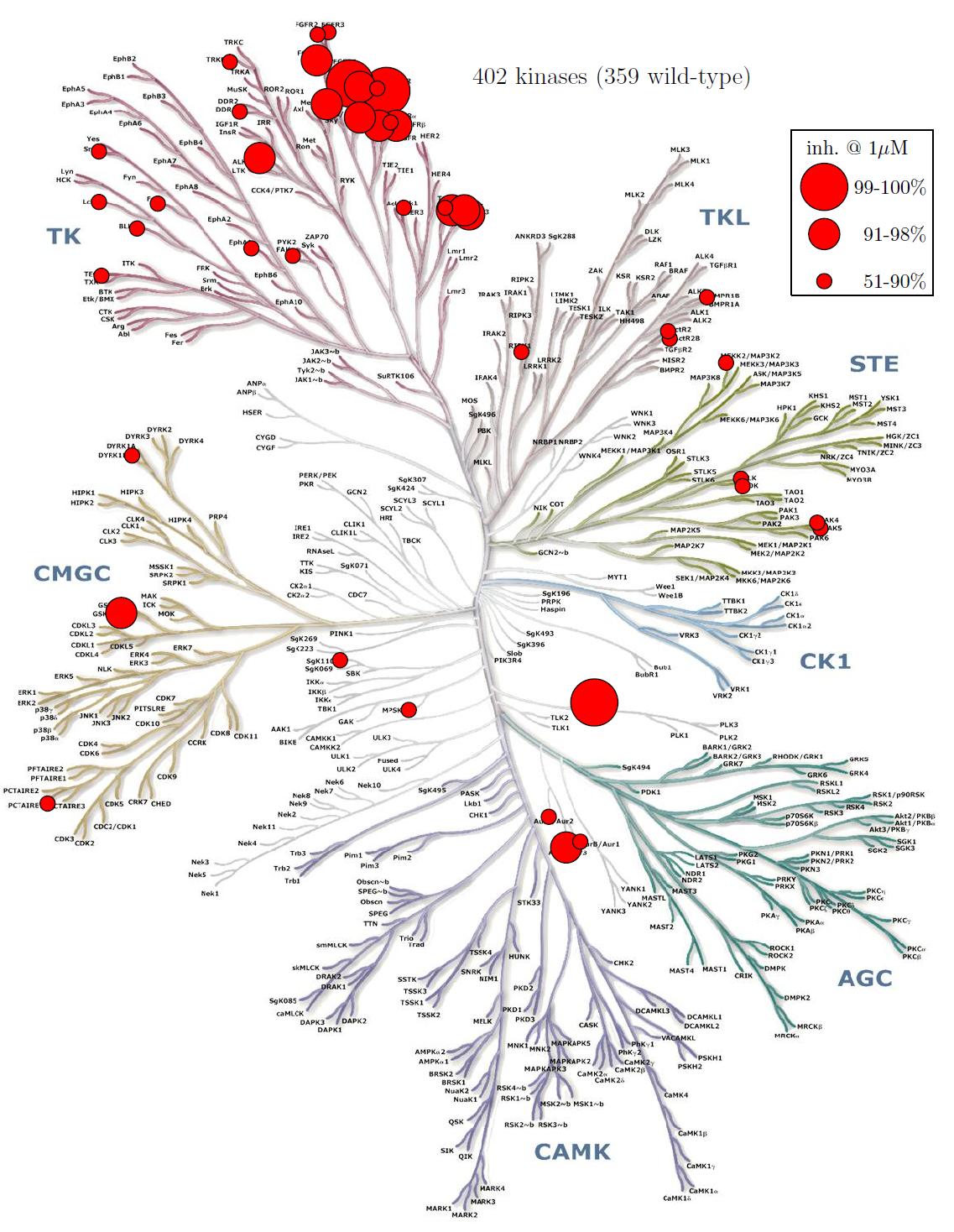


**FigureS1. Treespot map of R1498 (1 μM) under ATP Km.**

Supplement: Figure S1 — Treespot map of R1498 (1 µM) under ATP Km. The affinity of R1498 against 402 kinases was determined by KINOMEScan® (Ambit Biosciences, San Diego, CA). The inhibition was indicated with red dots of different size in a kinome tree. Bigger dot means strong inhibition. (DOC) [file pone.0065264.s001.doc]
