# Supplementary material for: Small Molecule R1498 as a Well-Tolerated and Orally Active Kinase Inhibitor for Hepatocellular Carcinoma and Gastric Cancer Treatment via Targeting Angiogenesis and Mitosis Pathways
Source: PLoS One. 2013 Jun 5;8(6):e65264. doi: 10.1371/journal.pone.0065264 (PMC3673949; doi:10.1371/journal.pone.0065264)
Supplement: Table S1 — Single dose PK profiles in multiple animal species. R1498 micronized active pharmaceutical ingredient (API) was formulated and dosed to multiple species per oral or intravenous injection. Plasma samples from various time points were collected and determined for R1498 concentration. (DOC) [file pone.0065264.s002.doc]

**Table S1. SDPK profiles in multiple animal species**

| **Species** | **Nude Mouse** | **Rat** | **Dog** | **Monkey** |
| --- | --- | --- | --- | --- |
| **Cl (mL/min/kg)** | 6.3 | 5.6 | 1.9 | 25.3 |
| **Vdss (L/kg)** | 2.0 | 1.6 | 2.7 | 2.1 |
| **T1/2 (h)** | 4.0 (iv 2 mg/kg) | 3.2  (iv 5 mg/kg) | 16.8  (iv 5 mg/kg) | 1.5 (iv 5 mg/kg) |
| **Oral BA (%)** | 44-92  (10-100 mg/kg) | 9-44 (25-400 mg/kg) | 32-45 (50-100 mg/kg) | 4  (12.5 mg/kg) |

*Micronized active pharmaceutical ingredient (API) + 1% Klucel LF + 0.1~0.3% Tween80 suspension formulation.
