# Supplementary material for: Small Molecule R1498 as a Well-Tolerated and Orally Active Kinase Inhibitor for Hepatocellular Carcinoma and Gastric Cancer Treatment via Targeting Angiogenesis and Mitosis Pathways
Source: PLoS One. 2013 Jun 5;8(6):e65264. doi: 10.1371/journal.pone.0065264 (PMC3673949; doi:10.1371/journal.pone.0065264)
Supplement: Table S2 — Efficacy of R1498 and sorafenib or cyclophosphamide on xenograft models of Chinese cancers. R1498, sorafenib and cyclophosphamide were tested on multiple xenografts with indicated schedules. (DOC) [file pone.0065264.s003.doc]

**Table S2. Efficacy of R1498 and sorafenib or cyclophosphamide on xenograft models of Chinese cancers**

| **Cancer type** | **Model** | **Group** | **Dosage^1^**  **(mg/kg)** | **Final TGI%^2^** | **Tumor Regression^3^** | **Body weight changes%^4^** |
| --- | --- | --- | --- | --- | --- | --- |
| **Chinese HCC** | **BEL-7402** | **R1498** | 25 | 102 | 8/10 | 2 |
|  |  | **Sorafenib** | 25 | 83 | 1/10 | -16 |
|  | **HCCLM3** | **R1498** | 12.5 | 81 | 0/10 | 15.3 |
|  |  | **Sorafenib** | 12.5 | 71 | 0/10 | 4.4 |
|  | **BEL-7404** | **R1498** | 12.5 | 90 | 0/10 | -1.9 |
|  |  | **Sorafenib** | 12.5 | 75 | 0/10 | -4.2 |
| **Chinese GC** | **BGC-823** | **R1498** | 25 | 94 | 0/10 | 2 |
|  |  | **Sorafenib** | 25 | 82 | 0/10 | -3.5 |
|  | **MGC-803** | **R1498** | 25 | 99 | 5/10 | 14 |
|  |  | **Sorafenib** | 25 | 92 | 2/10 | -2.4 |
|  | **HGC-27** | **R1498** | 25 | 85 | 0/10 | 6 |
|  |  | **Sorafenib** | 25 | 83 | 0/10 | -5.5 |
|  | **SGC-7901** | **R1498** | 25 | 100 | 5/10 | 2 |
|  |  | **Sorafenib** | 25 | 95 | 2/10 | -18 |
| **Chinese NPC** | **CNE-2** | **R1498** | 25 | 90 | 0/10 | 16.3 |
|  |  | **Cyclophosphamide** | 100 | 58 | 0/10 | -2.1 |

1. Twice a day per oral gavage;
2. On the day of study termination;
3. The tumor regression of individual mouse was defined as tumor volume when treatment finished was less than the tumor volume when treatment initiated;
4. Based on comparison of body weight of individual mouse before and after treatment.
