# Supplementary material for: Small Molecule R1498 as a Well-Tolerated and Orally Active Kinase Inhibitor for Hepatocellular Carcinoma and Gastric Cancer Treatment via Targeting Angiogenesis and Mitosis Pathways
Source: PLoS One. 2013 Jun 5;8(6):e65264. doi: 10.1371/journal.pone.0065264 (PMC3673949; doi:10.1371/journal.pone.0065264)
Supplement: Table S3 — ED60/90 estimation from the R1498 exposure at steady state in tumors. The exposure in plasma and tumor at steady state were plot against tumor growth inhibiton rate to estimate the ED60/E90 doses. (DOC) [file pone.0065264.s004.doc]

**Table S3 ED60/90 estimation from the R1498 exposure at steady state in tumors.**

| **Model** | **Group** | **Final TGI%** | **Plasma AUC @ SS** | **Tumor AUC @ SS** |
| --- | --- | --- | --- | --- |
| **BEL-7402** | 3.125 mpk, bid, PO | 56 | 5378 | 2219 |
|  | 6.25 mpk, bid, PO | 69 | 12594 | 6713 |
|  | 12.5 mpk, bid, PO | 90 | 26110 | 23886 |
| **BEL-7404** | 3.125 mpk, bid, PO | 47 | 3558 | 9251 |
|  | 6.25 mpk, bid, PO | 67 | 6559 | 17558 |
|  | 12.5 mpk, bid, PO | 87 | 21564 | 37142 |
|  | 12.5 mpk, single dose, PO  (PK arm) | / | 15164 | 30996 |
| **BGC-823** | 6.25 mpk, bid, PO | 78 | 10833 | 9906 |
|  | 12.5 mpk, bid, PO | 88 | 31068 | 39971 |
|  | 25 mpk, bid, PO | 94 | 43277 | 70897 |
|  | 25 mpk, single dose, PO  (PK arm) | / | 73299 | 49597 |
| **SGC-7901** | 6.25 mpk, bid, PO | 69 | 11448 | 25249 |
|  | 12.5 mpk, bid, PO | 83 | 26856 | 48130 |
|  | 25 mpk, bid, PO | 95 | 47927 | 78767 |
|  | 25 mpk, single dose, PO  (PK arm) | / | 70067 | 147592 |
