# Supplementary material for: Small Molecule R1498 as a Well-Tolerated and Orally Active Kinase Inhibitor for Hepatocellular Carcinoma and Gastric Cancer Treatment via Targeting Angiogenesis and Mitosis Pathways
Source: PLoS One. 2013 Jun 5;8(6):e65264. doi: 10.1371/journal.pone.0065264 (PMC3673949; doi:10.1371/journal.pone.0065264)
Supplement: Table S4 — Metabolite profiles in pooled liver microsomes. Pooled liver microsomes were incubated with R1498 and the metabolites were identified to predict the most close species to human. (DOC) [file pone.0065264.s005.doc]

**Table S4.** Metabolite profiles in pooled liver microsomes

| **Pooled XLM MetID** | **Parent (M0)** | **M1 (M-14) Demethylation** | **M2 (M+16) Oxidation** | **Total M%** |
| --- | --- | --- | --- | --- |
| **Human** | 91.9 | 0.8 | 7.3 | 8.1 |
| **Mouse** | 94.8 | 3.3 | 1.9 | 5.2 |
| **Rat** | 90.8 | 3.9 | 5.3 | 9.2 |
| **Dog** | 99.0 | 1.0 | N.D. | 1.0 |
| **Monkey** | 43.3 | 42.4 | 14.3 | 56.7 |
