# Supplementary material for: Small Molecule R1498 as a Well-Tolerated and Orally Active Kinase Inhibitor for Hepatocellular Carcinoma and Gastric Cancer Treatment via Targeting Angiogenesis and Mitosis Pathways
Source: PLoS One. 2013 Jun 5;8(6):e65264. doi: 10.1371/journal.pone.0065264 (PMC3673949; doi:10.1371/journal.pone.0065264)
Supplement: Table S5 — Dose range finding (DRF)-tox and efficacious exposures of R1498. (DOC) [file pone.0065264.s006.doc]

**Table S5. Dose range finding (DRF)-tox^1^ and efficacious exposures of R1498**

| **Species** | **Dose**  **(mg/kg)** | **MTD** | **MTD AUC_0-24_**  **(ng*hr/ml)** | **MTD AUC**  **ED60 AUC^3^** | **MTD AUC**  **ED90 AUC^3^** |
| --- | --- | --- | --- | --- | --- |
| **Rat**  **Toxicity** | 0, 6.25, 50, 100 | 50 (F)  > 100 (M) | 211,000  > 155,000 | 21~42  > 15~31 | 8.1  > 6.0 |
| **Dog**  **Toxicity^2^** | 0, 1, 7.5, 15 | > 15 (F/M) | > 111,000 | > 11~22 | > 4.3 |

^1^ Non-GLP 2-week DRF study, bid dosing, p.o.

^2^ Histopathology results: no significant findings at all doses

^3^ Efficacy AUC: exposure in BEL-7402 xenograft model:

ED60 ≈ 5,000~10,000 ng.hr/mL ; ED90 ≈ 26,000 ng.hr/mL estimated from Table S2
